# Acknowledgement to Reviewers of *Foods* in 2017

**DOI:** 10.3390/foods7010010

**Published:** 2018-01-22

**Authors:** Foods Editorial Office

**Affiliations:** MDPI AG, St. Alban-Anlage 66, 4052 Basel, Switzerland

Peer review is an essential part in the publication process, ensuring that *Foods* maintains high quality standards for its published papers. In 2017, a total of 105 papers were published in the journal. Thanks to the cooperation of our reviewers, the median time to first decision was 19 days and the median time to publication was 46 days. The editors would like to express their sincere gratitude to the following reviewers for their time and dedication in 2017:

Abbeddou, SouheilaMarkiv, AnatoliyAbete, Maria CesarinaMarkowski, MarekAdhikari, KoushikMatsuura, BunzoAdler-Nissen, JensMattoo, AutarAiking, H.McAuley, Catherine M.Al-Fatimi, MohamedMcCrickerd, KeriAlmiro De Almeida, AgostinhoMenghini, LuigiAltemimi, AmmarMerah, OthmaneAndersen, Barbara VadMeyer, CarstenAngelino, DonatoMichalska, AnnaArnaud, ElodieMichel, MaximilianAsenstorfer, Robert E.Mine, YoshinoriBardají, EduardMirosa, MirandaBarker, Allen V.Mitchell, JohnBasile, TeodoraMogi, MasakiBautista Ortín, Ana BelénMorales, María Belén LópezBeretta, ClaudioMorán, LaraBertoni, GiuseppeMoreno, H.M.Besada, CristinaMujaffar, SaheedaBetoret, EsterMunang’andu, Hetron MweembaBhattacharyya, GargiMurkovic, MichaelBi, XiangNaik, SurabhiBianco, ArmandodorianoNakhauka, Ekesa BeatriceBirch, DawnNanjundaswamy, Ananda K.Birch, JohnNarita, NorihikoBishop-Hurley, SharonNey, Denise M.Boesveldt, SanneNicoletto, CarloBohn, TorstenNjintang, Nicolas YanouBosilevac, MickNolan, JohnBourquin, Leslie D.Nolvachai, YadaBowen, PhyllisNorambuena, FernandoBraakhuis, AndreaNúñez, OscarBrandt, KirstenObana, AkiraBroom, IainOgawa, YukiharuBrownlee, IainOlmedilla-Alonso, BegoñaBrudzynski, KatrinaOmar, Syed HarisBrychcy-Rajska, EwaPadalkar, MugdhaBuchanan, RobertPaeng, Ki-JungBurns, Duncan ThorburnPanzella, LuciaCabral, CéliaPapadimitriuou, KonstantinosCalingacion, MariafePapoutsis, KonstantinosCalín-Sánchez, ÁngelParisi, SalvatoreCanavari, MaurizioPasqualone, AntonellaCanini, AntonellaPatarata, LuísCaroprese, MariangelaPereira, Carlos DiasCenci-Goga, Beniamino T.Pereira, J.A.Chambers IV, EdgarPereira, Olívia R.Chand, SubhashPhilips, NeenaChen, GuangpingPiga, AntonioChen, GuibingPoblaciones, Maria J.Chen, Lih-GeengPognonec, PhilippeChen, Yung-HsiangPop, OanaChoudhury, MalayPost, Mark J.Cicero, ArrigoPouliot, YvesCoelhoso, IsabelPravst, IgorCooksey, KayProcopio, Jesús R.Cuccurullo, GennaroProestos, CharalamposDai, YuminRamalhosa, ElsaDel Caro, AlessandraRanzato, EliaDomingues, FernandaRasinger, Josef DanielDuong, Hai M.Rawson, AshishElias, MiguelReid, GregorEl-Seedi, Hesham R.Revilla, EugenioEsteves, EduardoRiu-Aumatell, MontserratFaleiro, Maria L.Rivero-Pérez, M. DoloresFarran-Codina, AndreuRizzolo, AnnaFarris, StefanoRodas-González, A.Fenelon, Mark A.Rodriguez-Couto, SusanaFernandez Morales, Francisco JesusRoh, ChanghyunFerreira, I.M.P.L.V.O.Roosen, JuttaFerruzzi, Mario G.Ros-McDonnell, LorenzoFigiel, AdamRusso, MarinaFilannino, PasqualeSaiano, FilippoFiore, AlbertoSakai, RyuichiFirmesse, OlivierSalaheen, SerajusFiszman, SusanaSalvo, AndreaFlamini, GuidoSamaniego Vaesken, María De LourdesFouladkhah, AliyarSantini, AntonelloFoutch, BrianSantos, Sónia A.O.Fraser, EvanŠčetar, MarioFuselli, FabioScherf, KatharinaGalata, EleniScherm, HaraldGalli, FrancescaSchmid, MarkusGan, Ren-YouSchnabel, UtaGassler, NikolausSchowalter, TimothyGetty, Kelly J.K.Seiquer, IsabelGhawi, Sameer KhalilSendra, EstherGiacosa, SimoneSepčić, KristinaGiamperi, LauraSerio, AnnalisaGiannakourou, Maria C.Shauna, ParkesGiaouris, EfstathiosShinomiya, KazufusaGiarratana, FilippoSirsat, SujataGiuffrida, DanieleSkau Nielsen, TinaGolmohamadi, AmirSoenen, StijnGoltz, Douglas M.Sommer, StephanGonzález-Pérez, José A.Soultos, NikolaosGow, Megan L.Sousa Fragoso, RuiGrazyna, JaworskaSpeck, MelanieHamlin, RobertSpeer, KarlHarasym, JoannaStaege, Martin S.Harding, IanStan, LauraHarmon, AlisonStockan, JenniHarper, JamesStoforos, Nikolaos G.Hayes, MariaSu, ChunmingHe, LiliSugahara, TakuyaHellwig, MichaelSugiura, M.Hempel, CorinnaSuleria, Hafiz AnsarHendriksma, HarmenSuzzi, GiovannaHernández, FranciscaSzumny, AntoniHerrero, BaudilioTaiti, CosimoHill, Arthur R.Tappi, SilviaHolick, MichaelTassou, ChrysoulaHuang, Guan-JhongTemple, NormanHutchins, AndreaTodaro, MassimoIbrahim, SalamTodd, EwenIossifidou, EleniTorrieri, ElenaJadeja, RaviTosi, PaolaJaeger, RalfTrafiałek, JoannaJames, TharappelTroise, Antonio DarioJandrić, ZoraTrueman, Clive N.Jaradat, AbdullahTrujillo, Antonio-JoséJay-Russell, MicheleTsopmo, ApollinaireJefferies, L.K.Tsouvaltzis, PavlosJensen, Annette NygaardUeta, IkuoJerković, IgorUlrich, DetlefJiang, LiuweiVan Der Kamp, Jan WillemJones, Jessica L.Van Zanten, Hannah H.E.Jordan, KieranVapaatalo, HeikkiJung, SamooelVarela-Moreiras, GregorioJuszczak, LesławVasconi, MauroKamiloglu, SenemVasile, CorneliaKatayama, ShigeruWaehrens, Sandra S.Keenan, MichaelWagner, AnikaKhouryieh, HannaWalker, ChristieKim, DaehwanWalsh, MarieKones, RichardWang, QinKourkoutas, YiannisWang, Selina C.Krasniewska, KarolinaWang, YunKrupa-Kozak, UrszulaWarriner, KeithKulozik, UlrichWelch, Aaron Z.Kuorwel, KuorwelWendin, KarinLavelli, VeraWestad, FrankLawrence, MarkWestenbrink, SusanneLeaver, Michael J.Wiczkowski, WieslawLidon, FernandoWright, AmandaLing, MauriceWu, Tzu-HuaLorenzi, VanninaXi, LinLücke, Friedrich-KarlYang, WeiLudwiczuk, AgnieszkaYang, Wei-hsiungLundén, JanneYang, Yu-ChiaoMadeddu, RobertoYperman, JanMaehre, Hanne K.Zabetakis, IoannisMahapatra, Ajit K.Zhang, YueMajumder, KaustavZhang, ZhenhuaMäkinen, SariZheng, JoleneMares, DarylZiuzina, DanaMarinangeli, Christopher P.F.Zucca, Paolo

